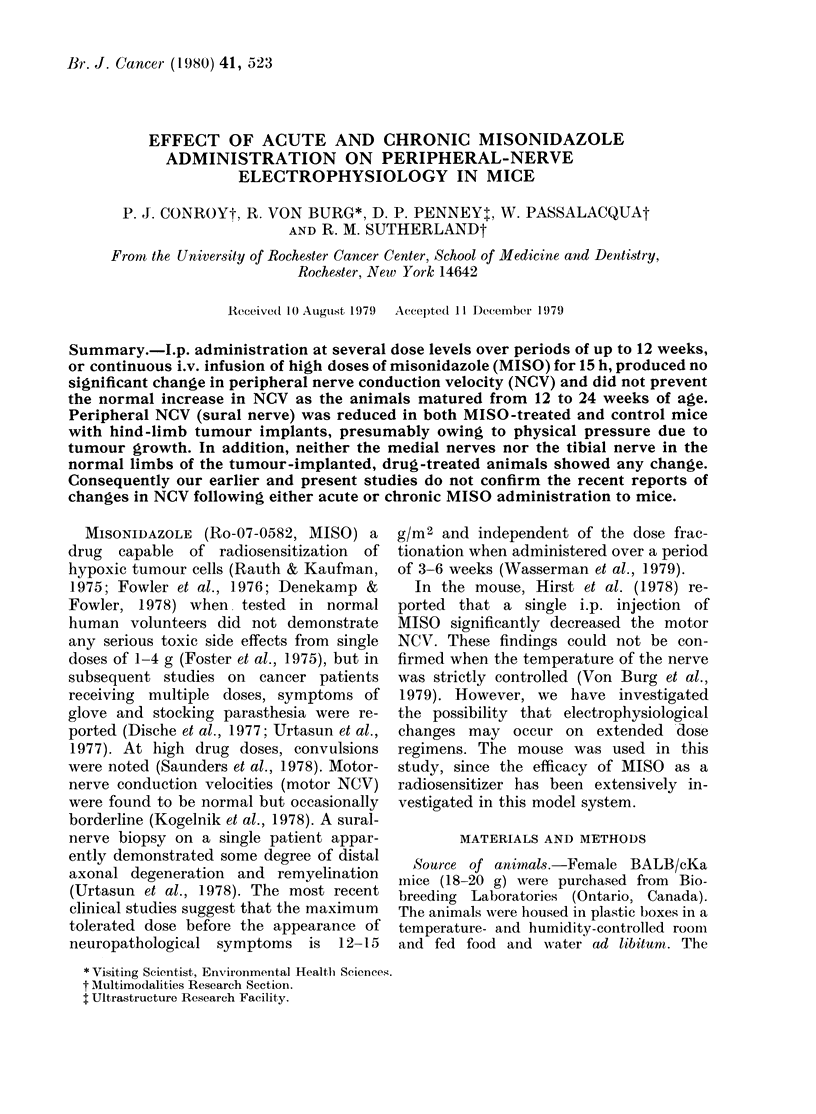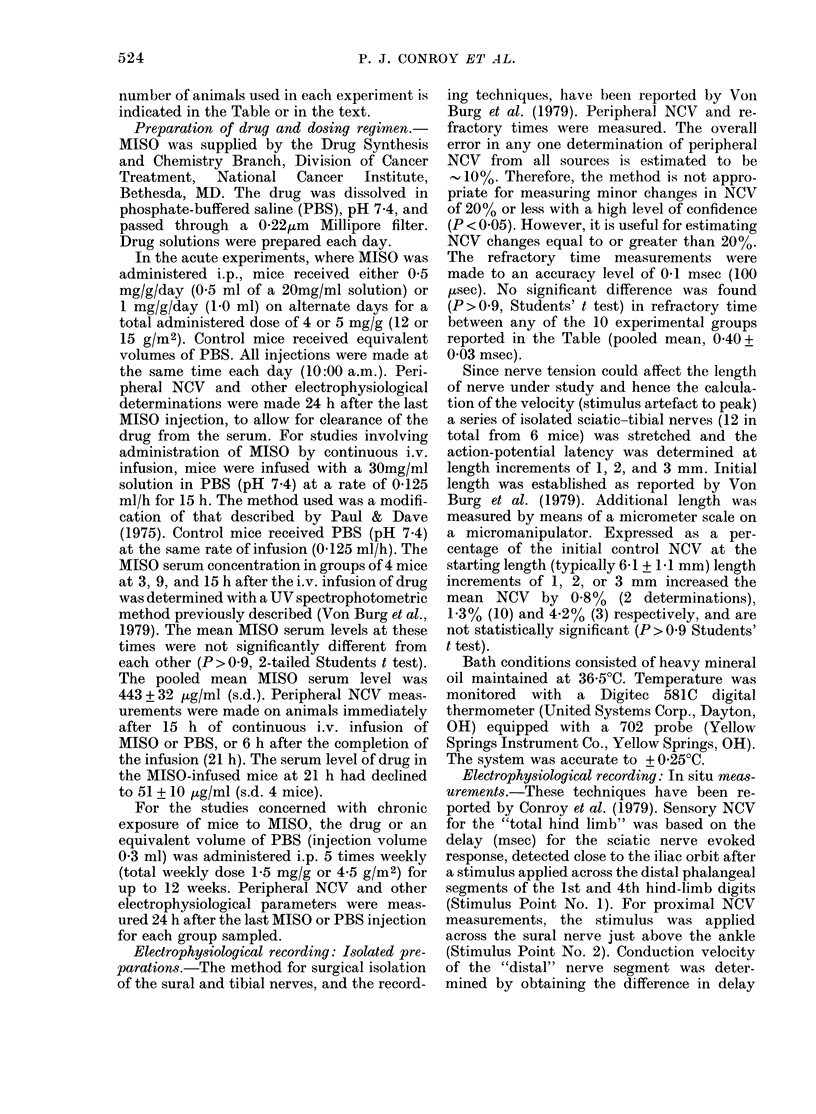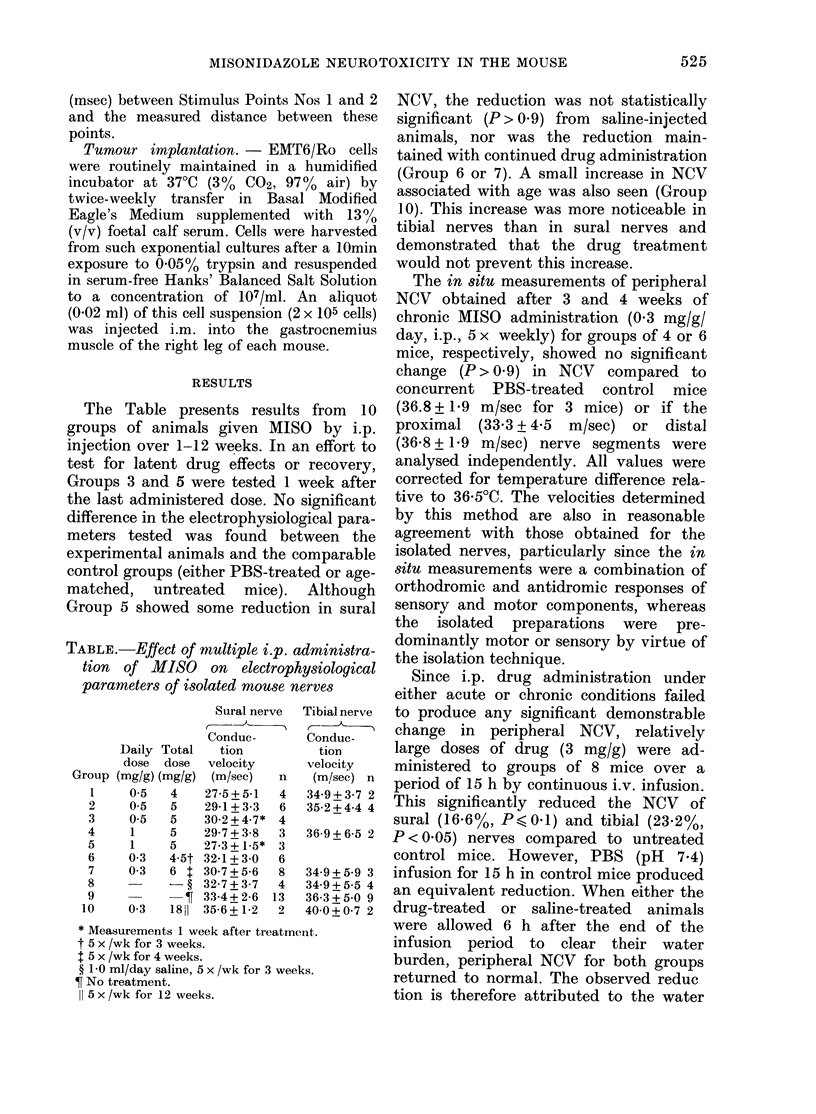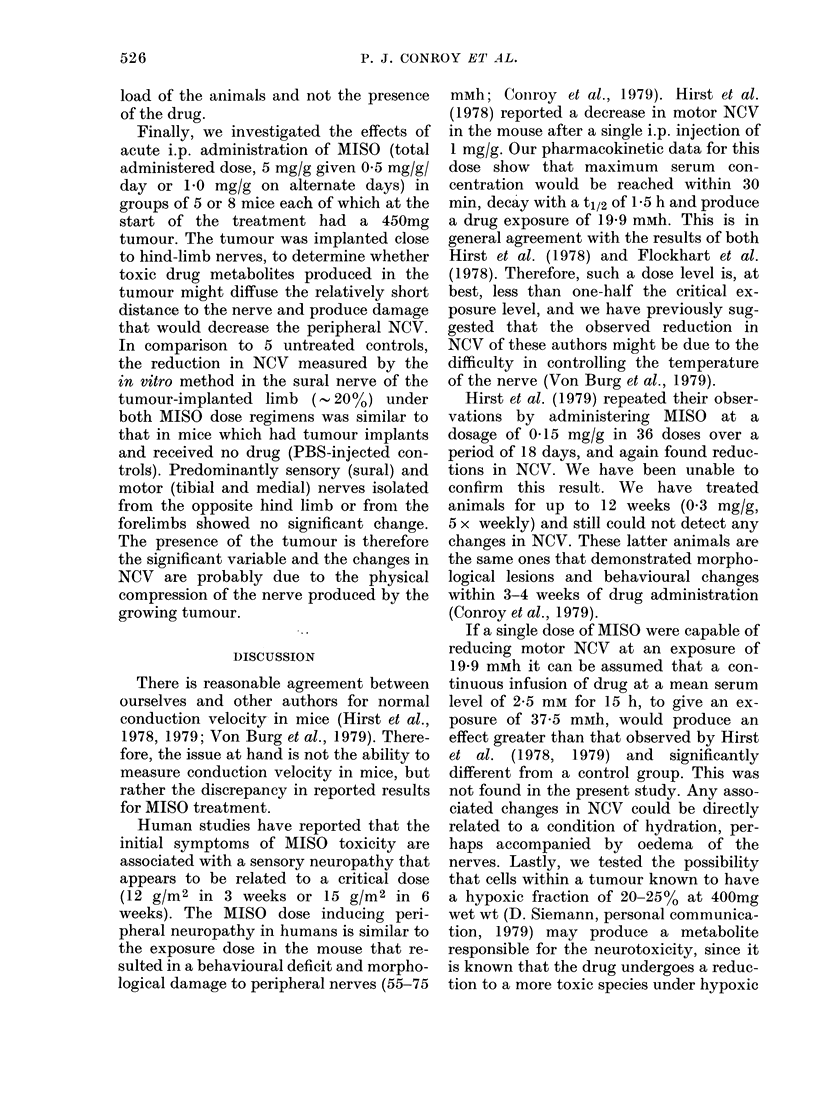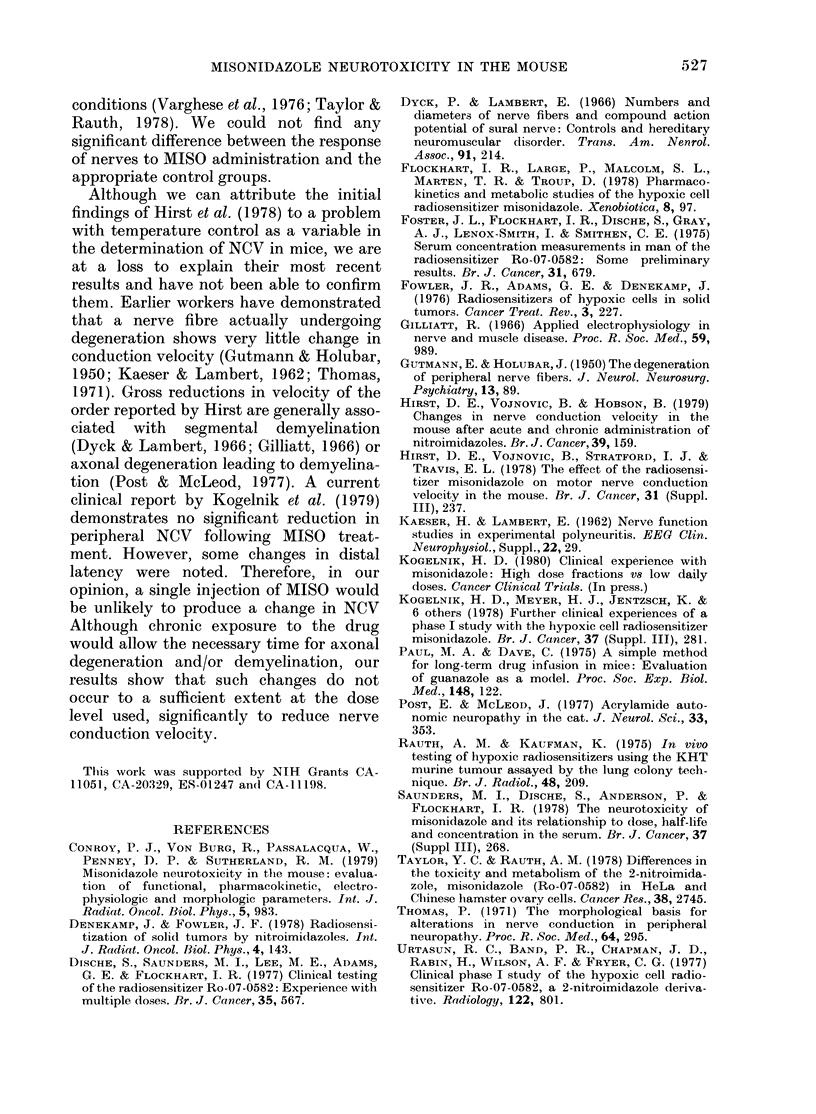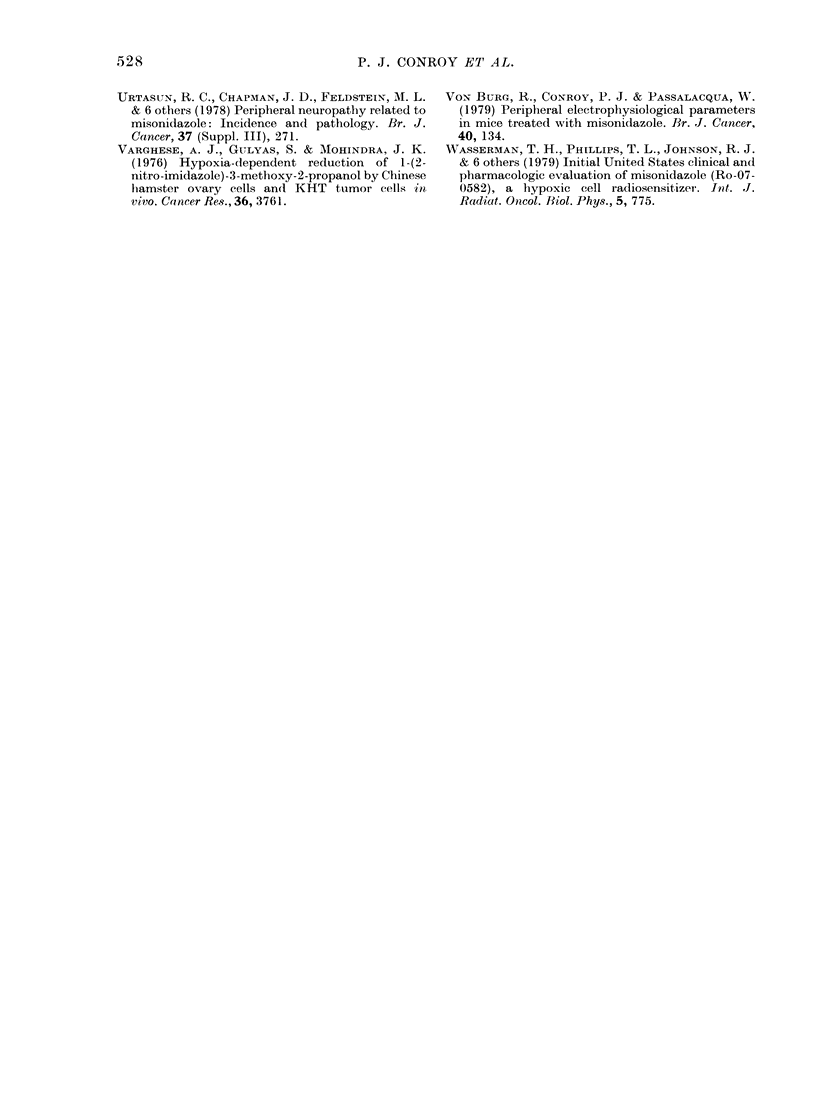# Effect of acute and chronic misonidazole administration on peripheral-nerve electrophysiology in mice.

**DOI:** 10.1038/bjc.1980.94

**Published:** 1980-04

**Authors:** P. J. Conroy, R. Von Burg, D. P. Penney, W. Passalacqua, R. M. Sutherland

## Abstract

I.p. administration at several dose levels over periods of up to 12 weeks, or continuous i.v. infusion of high doses of misonidazole (MISO) for 15 h, produced no significant change in peripheral nerve conduction velocity (NCV) and did not prevent the normal increase in NCV as the animals matured from 12 to 24 weeks of age. Peripheral NCV (sural nerve) was reduced in both MISO-treated and control mice with hind-limb tumour implants, presumably owing to physical pressure due to tumour growth. In addition, neither the medial nerves nor the tibial nerve in the normal limbs of the tumour-implanted, drug-treated animals showed any change. Consequently our earlier and present studies do not confirm the recent reports of changes in NCV following either acute or chronic MISO administration to mice.


*Br. J. Cancer* (1980) **41**, 523

### EFFECT OF ACUTE AND CHRONIC MISONIDAZOLE ADMINISTRATION ON PERIPHERAL-NERVE ELECTROPHYSIOLOGY IN MICE

P. J. CONROY†, R. VON BURG*, D. P. PENNEY‡, W. PASSALACQUA† AND R. M. SUTHERLAND†

*From the University of Rochester Cancer Center, School of Medicine and Dentistry, Rochester, New York* 14642

Received 10 August 1979 Accepted 11 December 1979

**Summary.—I.p. administration at several dose levels over periods of up to 12 weeks, or continuous i.v. infusion of high doses of misonidazole (MISO) for 15 h, produced no significant change in peripheral nerve conduction velocity (NCV) and did not prevent the normal increase in NCV as the animals matured from 12 to 24 weeks of age. Peripheral NCV (sural nerve) was reduced in both MISO-treated and control mice with hind-limb tumour implants, presumably owing to physical pressure due to tumour growth. In addition, neither the medial nerves nor the tibial nerve in the normal limbs of the tumour-implanted, drug-treated animals showed any change. Consequently our earlier and present studies do not confirm the recent reports of changes in NCV following either acute or chronic MISO administration to mice.**

MISONIDAZOLE (Ro-07-0582, MISO) a drug capable of radiosensitization of hypoxic tumour cells (Rauth & Kaufman, 1975; Fowler *et al.*, 1976; Denekamp & Fowler, 1978) when tested in normal human volunteers did not demonstrate any serious toxic side effects from single doses of 1–4 g (Foster *et al.*, 1975), but in subsequent studies on cancer patients receiving multiple doses, symptoms of glove and stocking parasthesia were reported (Dische *et al.*, 1977; Urtasun *et al.*, 1977). At high drug doses, convulsions were noted (Saunders *et al.*, 1978). Motor-nerve conduction velocities (motor NCV) were found to be normal but occasionally borderline (Kogelnik *et al.*, 1978). A sural-nerve biopsy on a single patient apparently demonstrated some degree of distal axonal degeneration and remyelination (Urtasun *et al.*, 1978). The most recent clinical studies suggest that the maximum tolerated dose before the appearance of neuropathological symptoms is 12–15 $g/m^2$ and independent of the dose fractionation when administered over a period of 3–6 weeks (Wasserman *et al.*, 1979).

In the mouse, Hirst *et al.* (1978) reported that a single i.p. injection of MISO significantly decreased the motor NCV. These findings could not be confirmed when the temperature of the nerve was strictly controlled (Von Burg *et al.*, 1979). However, we have investigated the possibility that electrophysiological changes may occur on extended dose regimens. The mouse was used in this study, since the efficacy of MISO as a radiosensitizer has been extensively investigated in this model system.

### MATERIALS AND METHODS

*Source of animals.*—Female BALB/cKa mice (18–20 g) were purchased from Bio-breeding Laboratories (Ontario, Canada). The animals were housed in plastic boxes in a temperature- and humidity-controlled room and fed food and water *ad libitum*. The

* Visiting Scientist, Environmental Health Sciences.

† Multimodalities Research Section.

‡ Ultrastructure Research Facility.

number of animals used in each experiment is indicated in the Table or in the text.

*Preparation of drug and dosing regimen.*—MISO was supplied by the Drug Synthesis and Chemistry Branch, Division of Cancer Treatment, National Cancer Institute, Bethesda, MD. The drug was dissolved in phosphate-buffered saline (PBS), pH 7·4, and passed through a 0·22$\mu$m Millipore filter. Drug solutions were prepared each day.

In the acute experiments, where MISO was administered i.p., mice received either 0·5 mg/g/day (0·5 ml of a 20mg/ml solution) or 1 mg/g/day (1·0 ml) on alternate days for a total administered dose of 4 or 5 mg/g (12 or 15 g/m$^2$). Control mice received equivalent volumes of PBS. All injections were made at the same time each day (10:00 a.m.). Peripheral NCV and other electrophysiological determinations were made 24 h after the last MISO injection, to allow for clearance of the drug from the serum. For studies involving administration of MISO by continuous i.v. infusion, mice were infused with a 30mg/ml solution in PBS (pH 7·4) at a rate of 0·125 ml/h for 15 h. The method used was a modification of that described by Paul & Dave (1975). Control mice received PBS (pH 7·4) at the same rate of infusion (0·125 ml/h). The MISO serum concentration in groups of 4 mice at 3, 9, and 15 h after the i.v. infusion of drug was determined with a UV spectrophotometric method previously described (Von Burg *et al.*, 1979). The mean MISO serum levels at these times were not significantly different from each other ($P > 0·9$, 2-tailed Students *t* test). The pooled mean MISO serum level was $443 \pm 32$ $\mu$g/ml (s.d.). Peripheral NCV measurements were made on animals immediately after 15 h of continuous i.v. infusion of MISO or PBS, or 6 h after the completion of the infusion (21 h). The serum level of drug in the MISO-infused mice at 21 h had declined to $51 \pm 10$ $\mu$g/ml (s.d. 4 mice).

For the studies concerned with chronic exposure of mice to MISO, the drug or an equivalent volume of PBS (injection volume 0·3 ml) was administered i.p. 5 times weekly (total weekly dose 1·5 mg/g or 4·5 g/m$^2$) for up to 12 weeks. Peripheral NCV and other electrophysiological parameters were measured 24 h after the last MISO or PBS injection for each group sampled.

*Electrophysiological recording: Isolated preparations.*—The method for surgical isolation of the sural and tibial nerves, and the recording techniques, have been reported by Von Burg *et al.* (1979). Peripheral NCV and refractory times were measured. The overall error in any one determination of peripheral NCV from all sources is estimated to be ~10%. Therefore, the method is not appropriate for measuring minor changes in NCV of 20% or less with a high level of confidence ($P < 0·05$). However, it is useful for estimating NCV changes equal to or greater than 20%. The refractory time measurements were made to an accuracy level of 0·1 msec (100 $\mu$sec). No significant difference was found ($P > 0·9$, Students' *t* test) in refractory time between any of the 10 experimental groups reported in the Table (pooled mean, $0·40 \pm 0·03$ msec).

Since nerve tension could affect the length of nerve under study and hence the calculation of the velocity (stimulus artefact to peak) a series of isolated sciatic–tibial nerves (12 in total from 6 mice) was stretched and the action-potential latency was determined at length increments of 1, 2, and 3 mm. Initial length was established as reported by Von Burg *et al.* (1979). Additional length was measured by means of a micrometer scale on a micromanipulator. Expressed as a percentage of the initial control NCV at the starting length (typically $6·1 \pm 1·1$ mm) length increments of 1, 2, or 3 mm increased the mean NCV by 0·8% (2 determinations), 1·3% (10) and 4·2% (3) respectively, and are not statistically significant ($P > 0·9$ Students' *t* test).

Bath conditions consisted of heavy mineral oil maintained at 36·5°C. Temperature was monitored with a Digitec 581C digital thermometer (United Systems Corp., Dayton, OH) equipped with a 702 probe (Yellow Springs Instrument Co., Yellow Springs, OH). The system was accurate to $\pm 0·25$°C.

*Electrophysiological recording:* In situ *measurements.*—These techniques have been reported by Conroy *et al.* (1979). Sensory NCV for the "total hind limb" was based on the delay (msec) for the sciatic nerve evoked response, detected close to the iliac orbit after a stimulus applied across the distal phalangeal segments of the 1st and 4th hind-limb digits (Stimulus Point No. 1). For proximal NCV measurements, the stimulus was applied across the sural nerve just above the ankle (Stimulus Point No. 2). Conduction velocity of the "distal" nerve segment was determined by obtaining the difference in delay

(msec) between Stimulus Points Nos 1 and 2 and the measured distance between these points.

*Tumour implantation.* — EMT6/Ro cells were routinely maintained in a humidified incubator at 37°C (3% $CO_2$, 97% air) by twice-weekly transfer in Basal Modified Eagle's Medium supplemented with 13% (v/v) foetal calf serum. Cells were harvested from such exponential cultures after a 10min exposure to 0·05% trypsin and resuspended in serum-free Hanks' Balanced Salt Solution to a concentration of $10^7$/ml. An aliquot (0·02 ml) of this cell suspension ($2 \times 10^5$ cells) was injected i.m. into the gastrocnemius muscle of the right leg of each mouse.

### RESULTS

The Table presents results from 10 groups of animals given MISO by i.p. injection over 1–12 weeks. In an effort to test for latent drug effects or recovery, Groups 3 and 5 were tested 1 week after the last administered dose. No significant difference in the electrophysiological parameters tested was found between the experimental animals and the comparable control groups (either PBS-treated or age-matched, untreated mice). Although Group 5 showed some reduction in sural NCV, the reduction was not statistically significant ($P > 0·9$) from saline-injected animals, nor was the reduction maintained with continued drug administration (Group 6 or 7). A small increase in NCV associated with age was also seen (Group 10). This increase was more noticeable in tibial nerves than in sural nerves and demonstrated that the drug treatment would not prevent this increase.

TABLE.—*Effect of multiple i.p. administration of MISO on electrophysiological parameters of isolated mouse nerves*

| | | | Sural nerve | | Tibial nerve | |
|---|---|---|---|---|---|---|
| Group | Daily dose (mg/g) | Total dose (mg/g) | Conduction velocity (m/sec) | n | Conduction velocity (m/sec) | n |
| 1 | 0·5 | 4 | 27·5 ± 5·1 | 4 | 34·9 ± 3·7 | 2 |
| 2 | 0·5 | 5 | 29·1 ± 3·3 | 6 | 35·2 ± 4·4 | 4 |
| 3 | 0·5 | 5 | 30·2 ± 4·7* | 4 | | |
| 4 | 1 | 5 | 29·7 ± 3·8 | 3 | 36·9 ± 6·5 | 2 |
| 5 | 1 | 5 | 27·3 ± 1·5* | 3 | | |
| 6 | 0·3 | 4·5† | 32·1 ± 3·0 | 6 | | |
| 7 | 0·3 | 6 ‡ | 30·7 ± 5·6 | 8 | 34·9 ± 5·9 | 3 |
| 8 | — | — § | 32·7 ± 3·7 | 4 | 34·9 ± 5·5 | 4 |
| 9 | — | — ¶ | 33·4 ± 2·6 | 13 | 36·3 ± 5·0 | 9 |
| 10 | 0·3 | 18‖ | 35·6 ± 1·2 | 2 | 40·0 ± 0·7 | 2 |

* Measurements 1 week after treatment.

† 5 × /wk for 3 weeks.

‡ 5 × /wk for 4 weeks.

§ 1·0 ml/day saline, 5 × /wk for 3 weeks.

¶ No treatment.

‖ 5 × /wk for 12 weeks.

The *in situ* measurements of peripheral NCV obtained after 3 and 4 weeks of chronic MISO administration (0·3 mg/g/day, i.p., 5 × weekly) for groups of 4 or 6 mice, respectively, showed no significant change ($P > 0·9$) in NCV compared to concurrent PBS-treated control mice (36.8 ± 1·9 m/sec for 3 mice) or if the proximal (33·3 ± 4·5 m/sec) or distal (36·8 ± 1·9 m/sec) nerve segments were analysed independently. All values were corrected for temperature difference relative to 36·5°C. The velocities determined by this method are also in reasonable agreement with those obtained for the isolated nerves, particularly since the *in situ* measurements were a combination of orthodromic and antidromic responses of sensory and motor components, whereas the isolated preparations were predominantly motor or sensory by virtue of the isolation technique.

Since i.p. drug administration under either acute or chronic conditions failed to produce any significant demonstrable change in peripheral NCV, relatively large doses of drug (3 mg/g) were administered to groups of 8 mice over a period of 15 h by continuous i.v. infusion. This significantly reduced the NCV of sural (16·6%, $P \leqslant 0·1$) and tibial (23·2%, $P < 0·05$) nerves compared to untreated control mice. However, PBS (pH 7·4) infusion for 15 h in control mice produced an equivalent reduction. When either the drug-treated or saline-treated animals were allowed 6 h after the end of the infusion period to clear their water burden, peripheral NCV for both groups returned to normal. The observed reduc tion is therefore attributed to the water load of the animals and not the presence of the drug.

Finally, we investigated the effects of acute i.p. administration of MISO (total administered dose, 5 mg/g given 0·5 mg/g/day or 1·0 mg/g on alternate days) in groups of 5 or 8 mice each of which at the start of the treatment had a 450mg tumour. The tumour was implanted close to hind-limb nerves, to determine whether toxic drug metabolites produced in the tumour might diffuse the relatively short distance to the nerve and produce damage that would decrease the peripheral NCV. In comparison to 5 untreated controls, the reduction in NCV measured by the *in vitro* method in the sural nerve of the tumour-implanted limb ($\sim 20\%$) under both MISO dose regimens was similar to that in mice which had tumour implants and received no drug (PBS-injected controls). Predominantly sensory (sural) and motor (tibial and medial) nerves isolated from the opposite hind limb or from the forelimbs showed no significant change. The presence of the tumour is therefore the significant variable and the changes in NCV are probably due to the physical compression of the nerve produced by the growing tumour.

### DISCUSSION

There is reasonable agreement between ourselves and other authors for normal conduction velocity in mice (Hirst *et al.*, 1978, 1979; Von Burg *et al.*, 1979). Therefore, the issue at hand is not the ability to measure conduction velocity in mice, but rather the discrepancy in reported results for MISO treatment.

Human studies have reported that the initial symptoms of MISO toxicity are associated with a sensory neuropathy that appears to be related to a critical dose (12 $g/m^2$ in 3 weeks or 15 $g/m^2$ in 6 weeks). The MISO dose inducing peripheral neuropathy in humans is similar to the exposure dose in the mouse that resulted in a behavioural deficit and morphological damage to peripheral nerves (55–75 mMh; Conroy *et al.*, 1979). Hirst *et al.* (1978) reported a decrease in motor NCV in the mouse after a single i.p. injection of 1 mg/g. Our pharmacokinetic data for this dose show that maximum serum concentration would be reached within 30 min, decay with a $t_{1/2}$ of 1·5 h and produce a drug exposure of 19·9 mMh. This is in general agreement with the results of both Hirst *et al.* (1978) and Flockhart *et al.* (1978). Therefore, such a dose level is, at best, less than one-half the critical exposure level, and we have previously suggested that the observed reduction in NCV of these authors might be due to the difficulty in controlling the temperature of the nerve (Von Burg *et al.*, 1979).

Hirst *et al.* (1979) repeated their observations by administering MISO at a dosage of 0·15 mg/g in 36 doses over a period of 18 days, and again found reductions in NCV. We have been unable to confirm this result. We have treated animals for up to 12 weeks (0·3 mg/g, 5 × weekly) and still could not detect any changes in NCV. These latter animals are the same ones that demonstrated morphological lesions and behavioural changes within 3–4 weeks of drug administration (Conroy *et al.*, 1979).

If a single dose of MISO were capable of reducing motor NCV at an exposure of 19·9 mMh it can be assumed that a continuous infusion of drug at a mean serum level of 2·5 mM for 15 h, to give an exposure of 37·5 mMh, would produce an effect greater than that observed by Hirst *et al.* (1978, 1979) and significantly different from a control group. This was not found in the present study. Any associated changes in NCV could be directly related to a condition of hydration, perhaps accompanied by oedema of the nerves. Lastly, we tested the possibility that cells within a tumour known to have a hypoxic fraction of 20–25% at 400mg wet wt (D. Siemann, personal communication, 1979) may produce a metabolite responsible for the neurotoxicity, since it is known that the drug undergoes a reduction to a more toxic species under hypoxic conditions (Varghese *et al.*, 1976; Taylor & Rauth, 1978). We could not find any significant difference between the response of nerves to MISO administration and the appropriate control groups.

Although we can attribute the initial findings of Hirst *et al.* (1978) to a problem with temperature control as a variable in the determination of NCV in mice, we are at a loss to explain their most recent results and have not been able to confirm them. Earlier workers have demonstrated that a nerve fibre actually undergoing degeneration shows very little change in conduction velocity (Gutmann & Holubar, 1950; Kaeser & Lambert, 1962; Thomas, 1971). Gross reductions in velocity of the order reported by Hirst are generally associated with segmental demyelination (Dyck & Lambert, 1966; Gilliatt, 1966) or axonal degeneration leading to demyelination (Post & McLeod, 1977). A current clinical report by Kogelnik *et al.* (1979) demonstrates no significant reduction in peripheral NCV following MISO treatment. However, some changes in distal latency were noted. Therefore, in our opinion, a single injection of MISO would be unlikely to produce a change in NCV Although chronic exposure to the drug would allow the necessary time for axonal degeneration and/or demyelination, our results show that such changes do not occur to a sufficient extent at the dose level used, significantly to reduce nerve conduction velocity.

This work was supported by NIH Grants CA-11051, CA-20329, ES-01247 and CA-11198.

### REFERENCES

Conroy, P. J., Von Burg, R., Passalacqua, W., Penney, D. P. & Sutherland, R. M. (1979) Misonidazole neurotoxicity in the mouse: evaluation of functional, pharmacokinetic, electrophysiologic and morphologic parameters. *Int. J. Radiat. Oncol. Biol. Phys.*, **5**, 983.

Denekamp, J. & Fowler, J. F. (1978) Radiosensitization of solid tumors by nitroimidazoles. *Int. J. Radiat. Oncol. Biol. Phys.*, **4**, 143.

Dische, S., Saunders, M. I., Lee, M. E., Adams, G. E. & Flockhart, I. R. (1977) Clinical testing of the radiosensitizer Ro-07-0582: Experience with multiple doses. *Br. J. Cancer*, **35**, 567.

Dyck, P. & Lambert, E. (1966) Numbers and diameters of nerve fibers and compound action potential of sural nerve: Controls and hereditary neuromuscular disorder. *Trans. Am. Nenrol. Assoc.*, **91**, 214.

Flockhart, I. R., Large, P., Malcolm, S. L., Marten, T. R. & Troup, D. (1978) Pharmacokinetics and metabolic studies of the hypoxic cell radiosensitizer misonidazole. *Xenobiotica*, **8**, 97.

Foster, J. L., Flockhart, I. R., Dische, S., Gray, A. J., Lenox-Smith, I. & Smithen, C. E. (1975) Serum concentration measurements in man of the radiosensitizer Ro-07-0582: Some preliminary results. *Br. J. Cancer*, **31**, 679.

Fowler, J. R., Adams, G. E. & Denekamp, J. (1976) Radiosensitizers of hypoxic cells in solid tumors. *Cancer Treat. Rev.*, **3**, 227.

Gilliatt, R. (1966) Applied electrophysiology in nerve and muscle disease. *Proc. R. Soc. Med.*, **59**, 989.

Gutmann, E. & Holubar, J. (1950) The degeneration of peripheral nerve fibers. *J. Neurol. Neurosurg. Psychiatry*, **13**, 89.

Hirst, D. E., Vojnovic, B. & Hobson, B. (1979) Changes in nerve conduction velocity in the mouse after acute and chronic administration of nitroimidazoles. *Br. J. Cancer*, **39**, 159.

Hirst, D. E., Vojnovic, B., Stratford, I. J. & Travis, E. L. (1978) The effect of the radiosensitizer misonidazole on motor nerve conduction velocity in the mouse. *Br. J. Cancer*, **31** (Suppl. III), 237.

Kaeser, H. & Lambert, E. (1962) Nerve function studies in experimental polyneuritis. *EEG Clin. Neurophysiol.*, Suppl., **22**, 29.

Kogelnik, H. D. (1980) Clinical experience with misonidazole: High dose fractions *vs* low daily doses. *Cancer Clinical Trials.* (In press.)

Kogelnik, H. D., Meyer, H. J., Jentzsch, K. & 6 others (1978) Further clinical experiences of a phase I study with the hypoxic cell radiosensitizer misonidazole. *Br. J. Cancer*, **37** (Suppl. III), 281.

Paul, M. A. & Dave, C. (1975) A simple method for long-term drug infusion in mice: Evaluation of guanazole as a model. *Proc. Soc. Exp. Biol. Med.*, **148**, 122.

Post, E. & McLeod, J. (1977) Acrylamide autonomic neuropathy in the cat. *J. Neurol. Sci.*, **33**, 353.

Rauth, A. M. & Kaufman, K. (1975) *In vivo* testing of hypoxic radiosensitizers using the KHT murine tumour assayed by the lung colony technique. *Br. J. Radiol.*, **48**, 209.

Saunders, M. I., Dische, S., Anderson, P. & Flockhart, I. R. (1978) The neurotoxicity of misonidazole and its relationship to dose, half-life and concentration in the serum. *Br. J. Cancer*, **37** (Suppl III), 268.

Taylor, Y. C. & Rauth, A. M. (1978) Differences in the toxicity and metabolism of the 2-nitroimidazole, misonidazole (Ro-07-0582) in HeLa and Chinese hamster ovary cells. *Cancer Res.*, **38**, 2745.

Thomas, P. (1971) The morphological basis for alterations in nerve conduction in peripheral neuropathy. *Proc. R. Soc. Med.*, **64**, 295.

Urtasun, R. C., Band, P. R., Chapman, J. D., Rabin, H., Wilson, A. F. & Fryer, C. G. (1977) Clinical phase I study of the hypoxic cell radiosensitizer Ro-07-0582, a 2-nitroimidazole derivative. *Radiology*, **122**, 801.

URTASUN, R. C., CHAPMAN, J. D., FELDSTEIN, M. L. & 6 others (1978) Peripheral neuropathy related to misonidazole: Incidence and pathology. *Br. J. Cancer,* **37** (Suppl. III), 271.

VARGHESE, A. J., GULYAS, S. & MOHINDRA, J. K. (1976) Hypoxia-dependent reduction of 1-(2-nitro-imidazole)-3-methoxy-2-propanol by Chinese hamster ovary cells and KHT tumor cells *in vivo. Cancer Res.,* **36,** 3761.

VON BURG, R., CONROY, P. J. & PASSALACQUA, W. (1979) Peripheral electrophysiological parameters in mice treated with misonidazole. *Br. J. Cancer,* **40,** 134.

WASSERMAN, T. H., PHILLIPS, T. L., JOHNSON, R. J. & 6 others (1979) Initial United States clinical and pharmacologic evaluation of misonidazole (Ro-07-0582), a hypoxic cell radiosensitizer. *Int. J. Radiat. Oncol. Biol. Phys.,* **5,** 775.